# Independent Evaluation of the Rapid Scale-Up Program to Reduce Under-Five Mortality in Burkina Faso

**DOI:** 10.4269/ajtmh.15-0585

**Published:** 2016-03-02

**Authors:** Melinda Munos, Georges Guiella, Timothy Roberton, Abdoulaye Maïga, Adama Tiendrebeogo, Yvonne Tam, Jennifer Bryce, Banza Baya

**Affiliations:** Institute for International Programs, Johns Hopkins Bloomberg School of Public Health, Baltimore, Maryland; Institut Supérieur des Sciences de la Population, University of Ouagadougou, Ouagadougou, Burkina Faso; Université catholique de Louvain, Louvain-la-Neuve, Belgium; Institut National de la Statistique et de la Démographie, Ouagadougou, Burkina Faso

## Abstract

We conducted a prospective evaluation of the “Rapid Scale-Up” (RSU) program in Burkina Faso, focusing on the integrated community case management (iCCM) component of the program. We used a quasi-experimental design in which nine RSU districts were compared with seven districts without the program. The evaluation included documentation of program implementation, assessments of implementation and quality of care, baseline and endline coverage surveys, and estimation of mortality changes using the Lives Saved Tool. Although the program trained large numbers of community health workers, there were implementation shortcomings related to training, supervision, and drug stockouts. The quality of care provided to sick children was poor, and utilization of community health workers was low. Changes in intervention coverage were comparable in RSU and comparison areas. Estimated under-five mortality declined by 6.2% (from 110 to 103 deaths per 1,000 live births) in the RSU area and 4.2% (from 114 to 109 per 1,000 live births) in the comparison area. The RSU did not result in coverage increases or mortality reductions in Burkina Faso, but we cannot draw conclusions about the effectiveness of the iCCM strategy, given implementation shortcomings. The evaluation results highlight the need for greater attention to implementation of iCCM programs.

## Introduction

Over the course of the previous decade, there has been rapid and increasing investment in community case management (CCM) at country and global levels, prompted in part by the issuance of several World Health Organization (WHO)/United Nations Children's Fund (UNICEF) joint statements promoting CCM as a way to reduce mortality in children under five and increase equity.^1^,^2^ Donors have funded this scale-up through large initiatives like the Catalytic Initiative to Save a Million Lives^3^ and the Rapid Access Expansion Program,^4^ as well as smaller-scale projects, and many countries have now adopted policies allowing community health workers to treat conditions such as malaria, diarrhea, and suspected pneumonia.^5^

From 2008 to 2010, the Catalytic Initiative funded “proof of concept” evaluations in three countries (Burkina Faso, Malawi, and Mozambique; Mozambique was later dropped due to implementation challenges and Ethiopia was added instead) to show that proven interventions could be scaled up rapidly to reduce maternal, newborn, and child mortality. In each country, the Ministry of Health (MoH), together with a UN agency, planned and implemented a “Rapid Scale-Up” (RSU) of high-impact interventions that was evaluated prospectively by Johns Hopkins University together with in-country research partners.

Burkina Faso, which is ranked 181st of 187 countries on the Human Development Index,^6^ has declining but still high levels of mortality. The under-five mortality rate decreased from 186 per 1,000 live births in 2000 to 114 per 1,000 in 2010^7^; the maternal mortality ratio has fallen from 580 per 100,000 live births to 400 per 100,000 in 2013.^8^ The major causes of under-five deaths in Burkina in 2010 were infectious diseases for which effective treatments exist: malaria (23%), pneumonia (13%), and diarrhea (11%).^9^ Neonatal causes of death including infections, prematurity, and asphyxia together accounted for another 26% of under-five deaths in 2010.^9^ With RSU funding from the Catalytic Initiative and technical assistance from UNICEF, the MoH in Burkina Faso implemented a set of community- and facility-based interventions from 2009 to 2013 with the objectives of reducing the under-five mortality rate in the RSU area by 25% relative to baseline and of reaching specified targets for maternal, newborn, and child health (MNCH) intervention coverage in RSU areas (Table 1).^10^ No targets were established for maternal and neonatal mortality.

This article, focusing on the RSU in Burkina Faso, is one of three reports of full prospective evaluations of the implementation of the RSU. The objectives of the evaluation in Burkina Faso were to assess whether the program objectives^10^ were met and to assess the impact of the RSU strategy relative to ongoing activities in the rest of the country. The evaluation was conducted by an independent team of researchers from Johns Hopkins University as well as the Institut Supérieur des Sciences de la Population at the University of Ouagadougou, with assistance from the National Institute of Statistics and Demography (INSD) in Burkina Faso.

## Methods

### Program design and setting.

#### Setting.

**Figure 1.**
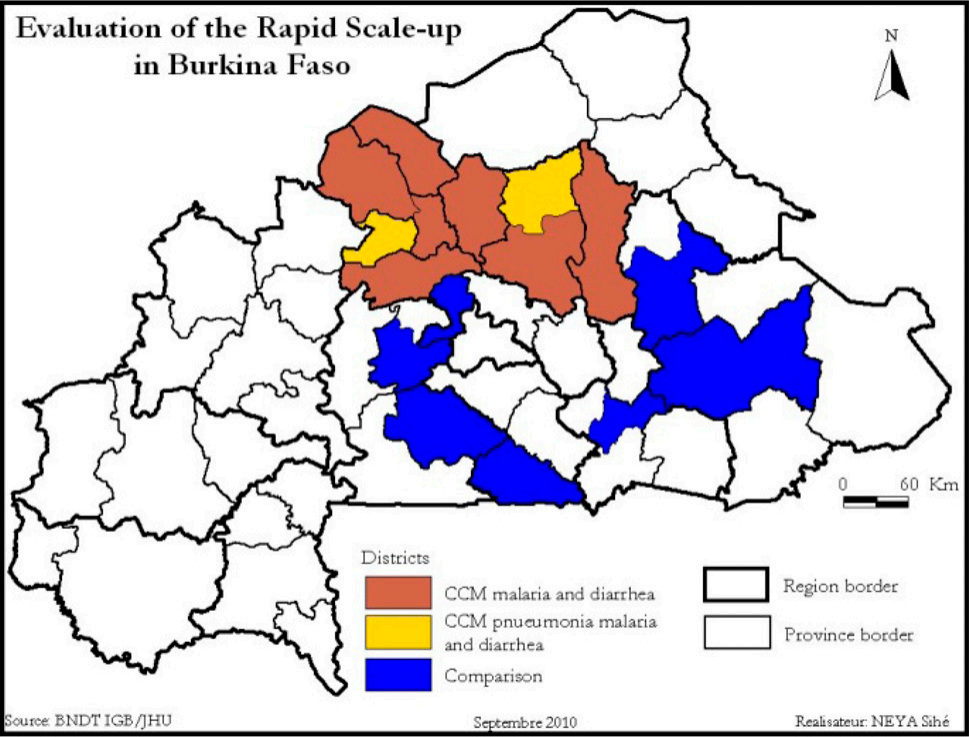
Program and comparison areas for the Rapid Scale-Up evaluation.

The RSU in Burkina Faso was implemented in nine health districts comprising the Nord and Centre-Nord regions of the country (Figure 1

). These regions were selected purposively by the MoH on the basis of high under-five mortality levels, capacity to absorb the project funds, and relative lack of investment by health and development partners.^10^ The independent evaluation team had no input in the selection of the program regions.

#### Program description.

The RSU in Burkina Faso included both community and facility components.^10^ The main aspect of the community component was the implementation of integrated community case management (iCCM) for diarrhea and malaria in seven program districts, and the implementation of iCCM for pneumonia, diarrhea, and malaria in two program districts. To enable implementation of this component, the MoH had to change its policy to allow community-based case management of diarrhea and malaria and to allow community-based treatment of pneumonia on a pilot basis in two districts.^11^ Other community-based activities included detection and referral of cases of acute malnutrition and promotion of healthy practices by community-based workers (called “Agents de santé à base communautaire” or ASBCs). A parallel national effort to implement malaria CCM, funded by the Global Fund and managed by Plan Burkina, was not integrated with the RSU.

The ASBCs who were responsible for the community-based component of the RSU were part of an existing cadre of lay volunteers in Burkina Faso.^11^ ASBCs were selected by the community in which they worked (two per village, one male and one female), were often illiterate, and received little to no preservice training upon being selected as ASBCs.^12^ iCCM-trained nurses at the local health center were responsible for supervising ASBCs in their catchment area; the number of ASBCs in a health facility catchment area in the program districts ranged from 2 to 48.^12^ The RSU planned to train all ASBCs within the program districts in iCCM and equip them with drug kits.^10^ Nurses were to supervise ASBCs in iCCM bimonthly (monthly in the areas implementing pneumonia CCM).^12^ ASBCs providing iCCM services were responsible for visiting the local health facility to restock their drug kits; they then could sell these drugs to community members at a markup to provide a small financial “motivation” for their work.^12^ Over the course of program implementation, the MoH made a number of policy changes affecting community health. These included the creation of a Directorate of Community Health (later absorbed into the Directorate of Health Promotion) and the development of a comprehensive policy on community health workers.^13^

The facility component of the RSU used project funds to support activities such as integrated management of childhood illness (IMCI); emergency obstetric and newborn care; emergency triage and treatment training for clinicians; and acquisition of commodities, such as delivery tables and bag and mask kits for hospitals, which were expected to reduce maternal, newborn, and under-five mortality.^10^ Funds were also used to support outreach activities such as child health days and insecticide-treated bednet (ITN) distribution campaigns.^10^ Because similar activities were ongoing throughout the country, the evaluation focused primarily on the implementation of iCCM, which was the one novel aspect of the project that might be expected to accelerate changes in coverage and mortality in the project districts, relative to other areas of the country.

### Evaluation design and conceptual framework.

#### Design.

**Figure 2.**
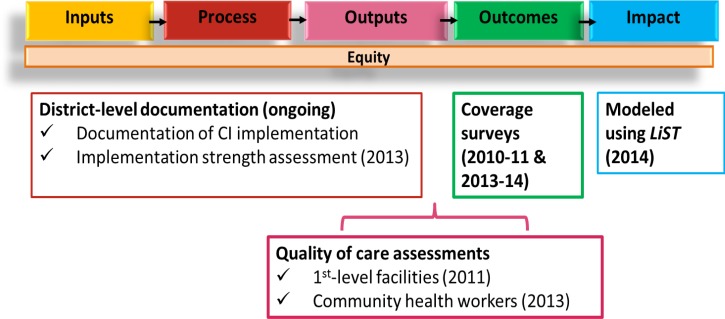
Framework for Rapid Scale-Up evaluation in Burkina Faso.

**Figure 3.**
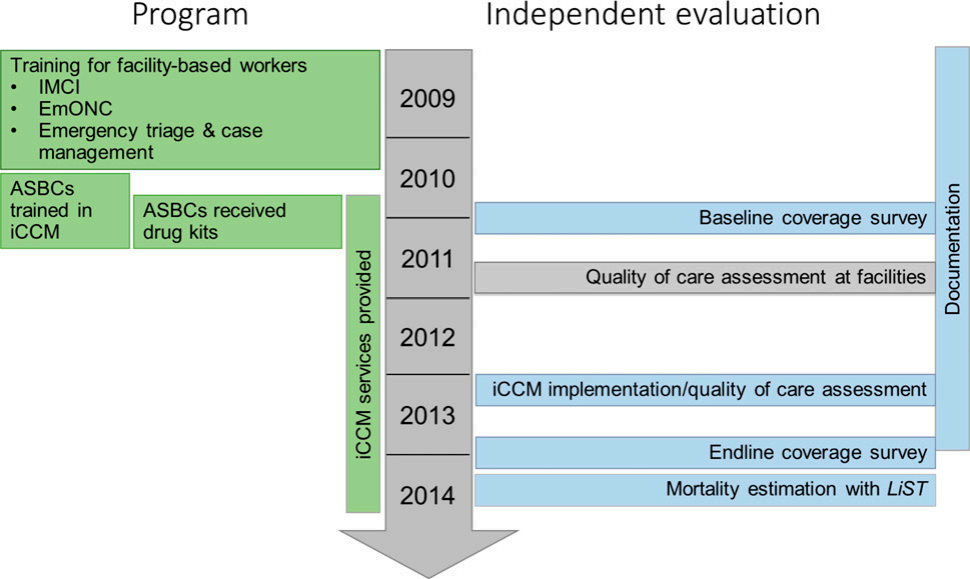
Evaluation timeline.

Because the program districts were not randomly selected, the evaluation design was restricted to a quasi-experimental approach with both pre-post and difference in differences analyses. The evaluation was guided by the common evaluation framework (Figure 2

).^14^ The evaluation timeline is shown in Figure 3

. The primary outcome for the evaluation was changes in intervention coverage in RSU and comparison areas, measured through baseline and endline coverage surveys. We did not measure mortality directly but rather modeled it based on changes in coverage using the Lives Saved Tool (LiST). We used measures of program strength and quality of care to inform our interpretation of changes in intervention coverage and mortality.

District-level documentation, baseline and endline coverage surveys, and mortality estimation with LiST were conducted in both project and comparison areas. The assessments of implementation strength and quality of care for iCCM were conducted only in the program area, primarily because there were no iCCM activities in comparison areas at the time of the assessments. Although comparison areas did have ASBCs, they were primarily responsible for health education and promotion activities, as well as some CCM of malaria through a Global Fund grant. The quality of care assessment for health facilities was funded and implemented separately by WHO with no input from the Independent Evaluation Team, and was conducted in the program area only.

#### Selection of comparison area.

A set of seven health districts was matched to the nine intervention districts using the following approach. The Independent Evaluation Team limited the pool of potential comparison districts to 25 districts, on the basis of ecological and cultural similarities to the project area. We defined matching criteria for six demographic and health systems indicators (Supplemental Web Annex 1, Table 1.1). We then identified 61,652 combinations of 7 districts from the pool of 25 candidate districts that satisfied our matching criteria. Of these, one set of seven districts was selected at random.^15^

### Data sources.

#### Documentation.

The evaluation's documentation system was separate from the program monitoring conducted by the MoH and was based on the abstraction of routine data in each district. Documentation data were collected prospectively from the third quarter of 2010 to the third quarter of 2013, and retrospectively for 2009 and the first and second quarters of 2010. We designed data abstraction forms covering facility and community-based activities, as well as outreach campaigns. Data were abstracted quarterly in each district in the program and comparison areas. Data were entered with CSPro 4.0^16^ (United States Census Bureau, Suitland, MD) and analyzed with SPSS version 17 (SPSS Inc., Chicago, IL).^17^

Qualitative interviews were conducted in each district every 6–12 months to collect data on contextual factors, including natural disasters, new projects, agricultural or economic development, and other factors that could affect MNCH in the districts. Interviews were conducted by two trained interviewers using a short interview guide and were recorded and transcribed. Transcriptions were coded using NVivo software^18^ and analyzed to look at prespecified themes.

We supplemented the collection of documentation data with the use of publicly available annual statistical handbooks produced by government ministries.^19^–^22^

#### Implementation strength and quality of care assessments.

Because districts had few data on iCCM available at district level, we conducted an assessment of the implementation of iCCM and quality of care provided by ASBCs in the program districts in February–May 2013.^12^ The methods used were based on an earlier study in Ethiopia,^23^ except that we provided ASBCs with a fully stocked drug kit to use during observations of sick child consultations, and we conducted in-depth interviews and focus groups with ASBCs and their supervisors as well as community members.

Briefly, the Independent Evaluation Team sampled 420 ASBCs across the nine program districts (30 in each of the districts without pneumonia CCM and 105 in each of the districts with pneumonia CCM) using systematic random sampling, of which 385 were found and agreed to participate. Data collectors interviewed each participating ASBC and inspected their drug kit and register. ASBC supervisors at the local health facility were also interviewed. Data collectors then observed up to two sick child consultations (for children aged 2–59 months) for each ASBC and recorded their observations in a questionnaire. They then conducted an exit interview with the caregiver accompanying the child. Finally, the sick children were reexamined by study team members who were experienced IMCI-trained clinicians, and the gold standard diagnoses and treatments for the child were recorded. Data were collected on Samsung Galaxy 2 and 3 smartphones (Samsung Electronics Co., Ltd., Seoul, Seoul Capital Area, South Korea) using Pendragon Forms software (Pendragon Software Corp., Chicago, IL).^24^ Data analysis was conducted in Stata, version 13 (StataCorp LP, College Station, TX).^25^

An assessment of the quality of care provided to sick children in health facilities was conducted in 2011 in the project districts. This assessment was funded and carried out separately by WHO in collaboration with the Institut pour la Recherche en Sciences de la Santé without involvement from the Independent Evaluation Team. The methods of this assessment were consistent with the WHO guidelines for quality of care assessments at health facilities,^26^ and the results have been published as a report.^27^ Briefly, 50 first-level health facilities were sampled across the nine RSU districts. Sampling was stratified by district with the number of facilities sampled in each district proportional to the number of first-level facilities in the district. Within each district, facilities were randomly sampled from a list of all first-level facilities. At each facility, the first five sick children aged less than 5 years presenting for care were enrolled. Data collectors observed the consultations of these children, and then interviewed the caregiver accompanying them. Children were then reexamined by a study team member who was a clinician to obtain the gold standard diagnosis and treatment. Data collectors also recorded information on training and supervision of health workers at the facility and on stocks of drugs and commodities. Results from the study report are presented in this article in support of the evaluation of the RSU program.

#### Coverage surveys.

Household coverage surveys were conducted in August 2010 to January 2011 (baseline) and November 2013 to March 2014 (endline) in both program and comparison areas. These surveys collected data on coverage of MNCH interventions, as well as on water, sanitation, and hygiene indicators.

For both surveys, two-stage cluster sampling was used, stratified by district. In each district, rural census enumeration areas (EAs) were sampled with probability proportional to size, with data on EA size provided by INSD based on the 2006 national census.^28^ Within each district, sampling was implicitly stratified by commune. Maps of the sampled EAs were updated and the households enumerated, and in each sampled EA, 30 households were selected using systematic sampling. All women aged 15–49 years and children aged less than 5 years in the sampled households were eligible for the survey.

The baseline survey sampled 2,000 households in each of the districts implementing pneumonia CCM, and 1,000 households in each of the remaining districts, for a total of 18,000 households. The endline survey sampled 3,000 households in each of the districts implementing pneumonia CCM, and 840 households in each of the remaining districts, for a total of 17,760 households. We oversampled in the districts implementing pneumonia CCM to provide sufficient power to detect the effect of this intervention on careseeking and treatment of pneumonia in these two districts relative to the comparison districts. These samples sizes were selected to provide 80% power to detect a difference of differences of 10–15 percentage points (pp) or better in intervention coverage levels between program and comparison areas. With these sample sizes, the evaluation also had 80% power to detect a difference in differences of at least 15 pp in pneumonia treatment coverage levels between the two districts implementing pneumonia CCM and the comparison districts.

Baseline data were collected on paper questionnaires, which were checked in the field. Questionnaires were double-entered using CSPro 4.0^16^ at the central Independent Evaluation Team office, data entry errors were reconciled, and logic checks were conducted. Endline data were collected on Samsung Galaxy S2 and S3 smartphones using Pendragon Forms VI software.^24^ Data were synchronized nightly with a secure Amazon webserver. Data were cleaned and logic checks run on a daily basis in Stata version 13,^25^ and any inconsistencies in the data were verified with field staff.

### Analysis.

#### Implementation and quality of care data.

Analysis of iCCM implementation and quality of care data was conducted in Stata versions 12 and 13.^25^,^29^ Data were weighted for unequal probability of sampling and for nonresponse. The Taylor linearization method was used to adjust standard errors for the effects of clustering.^30^

We calculated point estimates and 95% confidence intervals (CIs) using standard indicators where possible. Indicators were calculated for the RSU area as a whole, for districts without pneumonia CCM, and for districts with pneumonia CCM. For utilization, we had incomplete register data as only 70% of ASBCs could produce a register, and ASBC self-reports of the number of sick children seen were contradictory. We therefore calculated a range of possible utilization rates from these different data sources. We multiplied the mean number of sick children treated per ASBC per month based on register data by 12 months and by the estimated number of ASBCs providing iCCM services and divided the result by the estimated number of under-fives in the program area^21^ to produce an estimate of the mean number of contacts with an ASBC per child per year. We then did the same using the self-reported utilization data from ASBCs and reported both numbers.

We used the implementation and quality of care results to inform our interpretation of the data on changes in intervention coverage and mortality.

#### Coverage data.

We used standard coverage indicator definitions from the Multiple Indicator Cluster Surveys and the Commission on Information and Accountability in Women's and Children's Health.^31^,^32^ Point estimates and 95% CIs were calculated at baseline and endline for the program area (including districts with and without pneumonia CCM), comparison area, and each region and district within the program and comparison areas. We weighted data for unequal probability of sampling and for nonresponse, and used the Taylor series expansion method,^30^ which accounts for the effects of cluster sampling, to estimate the variance around point estimates.

#### Pre-post analysis.

For each coverage indicator, the percentage point change in coverage from baseline to endline was calculated by subtracting the baseline coverage value from the endline coverage value and calculating the 95% CI for the difference.

#### Difference in differences analysis.

The differences in differences analysis compared changes in intervention coverage in RSU areas and comparison areas. For indicators of careseeking for children with suspected pneumonia, and treatment of suspected pneumonia with antibiotics, we compared coverage changes in comparison areas to coverage changes in the two RSU districts implementing CCM for pneumonia. This analysis does not require that coverage levels in intervention and comparison areas be the same at baseline, but assumes that the RSU program is the only factor that could lead to changes in coverage in the RSU area. As we were evaluating the effectiveness of the RSU approach relative to the status quo, we also assumed that there were no major programs with the potential to affect MNCH intervention coverage in comparison areas. These assumptions are examined in the section on contextual factors.

The difference in differences in intervention coverage between program and comparison areas from baseline to endline was calculated by first computing the point estimates and standard errors for coverage indicators at baseline and endline in RSU and comparison areas, as described above. We then computed the point estimate and 95% CI for the linear combination of these indicators, subtracting the coverage change from baseline to endline in the comparison area from the coverage change from baseline to endline in the RSU area. We made no adjustments for multiple comparisons.

#### Mortality analysis.

We estimated changes in mortality using LiST.^33^ LiST is a module within the Spectrum software application that brings demographic, cause of death, and intervention coverage data together with the best available estimates of intervention effectiveness to estimate the effect on mortality produced by changes in coverage. LiST analyses make the assumption that changes in intervention coverage drive changes in mortality, and that the effects of more distal factors such as education or poverty on mortality are mediated by changes in intervention coverage.

We used LiST to produce four models of mortality change (overall program area, program districts with pneumonia CCM, program districts without pneumonia CCM, and comparison area) from 2010 to 2013. Demographic data for each district were taken from the MoH's annual statistical handbook,^20^,^21^ which provides population projections from the most recent census. Baseline mortality levels and cause of death data were estimated for each area by adjusting the national mortality and cause of death data^9^ using the baseline levels of intervention coverage in each area. Coverage data were taken from the baseline and endline coverage surveys.

At present, there is no agreed-upon approach for computing uncertainty bounds around LiST mortality estimates because of challenges in determining the extent to which errors and biases in the many different inputs are correlated. However, we conducted a sensitivity analysis by producing two additional projections, one using the upper bounds of the 95% CIs around the LiST intervention effectiveness estimates and one using the lower bounds. This produced a range of possible mortality reductions, which we report in the results. A similar approach was used by Bhutta and others^34^ in the recent Every Newborn Series.

### Ethical review.

Ethical approval for the evaluation was obtained from the Johns Hopkins Bloomberg School of Public Health Institutional Review Board (IRB) (IRB2590 and IRB3909) and from the Burkina Faso National Ethics Committee for Health Research (DELIBERATION No. 2009-67 and DELIBERATION No. 2012-10-69).

### Role of the funding source.

The evaluation sponsor had no role in the design of the evaluation; in the collection, analysis, or interpretation of data; in the writing of the report; or in the decision to submit the article for publication.

## Results

Baseline values for key variables for each arm of the evaluation are shown in Supplemental Web Annex 1, Table 2.1. There were no significant differences between the RSU and comparison districts at baseline, except that coverage of oral rehydration solution (ORS) for diarrhea was significantly higher in RSU districts relative to comparison districts.

### Intensity and quality of program implementation.

Initial program implementation appeared strong, with 3,399 ASBCs trained in 2010 (5.94 per 1,000 children under 5 years), increasing to 4,012 by 2013 (7.39 per 1,000 children under 5 years). In addition, 99% of ASBCs providing iCCM services in 2013 reported receiving an initial drug kit (Table 2). However, there were weaknesses in iCCM training: written training materials were used even for illiterate ASBCs, only 52% of ASBCs providing iCCM services were trained with clinical practice, the reported size of the training groups ranged from 1 to 150 (median 20), and a cascade training approach was used in which central-level staff trained district staff, and district staff then either conducted training themselves, or more frequently trained nurses in health facilities to conduct the training of CHWs. Training of district staff and nurses was conducted in 2009–10, while training of ASBCs was primarily conducted in 2010 (and in one district in 2011).

With respect to ongoing implementation, we observed that in 2013, only 64% of ASBCs having received iCCM training reported providing iCCM services in the previous 12 months. Further, only 38% of ASBCs providing iCCM services had been supervised in the past 3 months and only 9% had a fully stocked drug kit. A frequent problem in health facilities was the absence of IMCI-trained personnel: During the health facility assessment, only 62% of health facilities had at least one IMCI-trained health worker in the facility on the day of the assessment. Further, only 18% of health facilities had all essential oral medications in stock on the day of the visit.

We observed poor quality of care for sick children in both health facilities and the community (Tables 2 and 3). At community level, all aspects of case management, including assessment, classification, treatment, and counseling were poor, with only 36% of sick children correctly treated by ASBCs. Although health workers in facilities correctly managed 91% of children with uncomplicated malaria, only 18% of children with diarrhea and 34% of children with pneumonia were correctly managed.

Based on data from ASBC registers (which were available for 70% of ASBCs), mean utilization was two sick children/ASBC/month. Based on ASBC self-reports of utilization, the median time since the ASBC's last sick child consultation was 1 week, and mean utilization was 12 sick children/ASBC/month. Assuming 4,012 ASBCs in 2013, of which 64% were providing iCCM services and 572,238 under-fives,^21^ we estimated a utilization range of 0.11–0.65 ASBC contacts per child per year.

### Coverage changes.

We observed shifts in the coverage of careseeking for fever and the sources from which care was sought (Table 4 and Supplemental Web Annex 2 [Tables 2.9, 2.10, 2.12, 2.13, 2.15, 2.16]). Careseeking for fever from health facilities decreased by 9.7 pp (95% CI: −14.1 to −5.2) from baseline to endline, while careseeking from ASBCs increased by 2.6 pp (95% CI: 0.7–4.6). Careseeking for suspected pneumonia from ASBCs increased from 5.5 to 10.2%, a 4.6 pp increase (95% CI: −3.0 to 12.3), but the CIs were very wide. We saw little change in careseeking for diarrhea.

**Figure 4.**
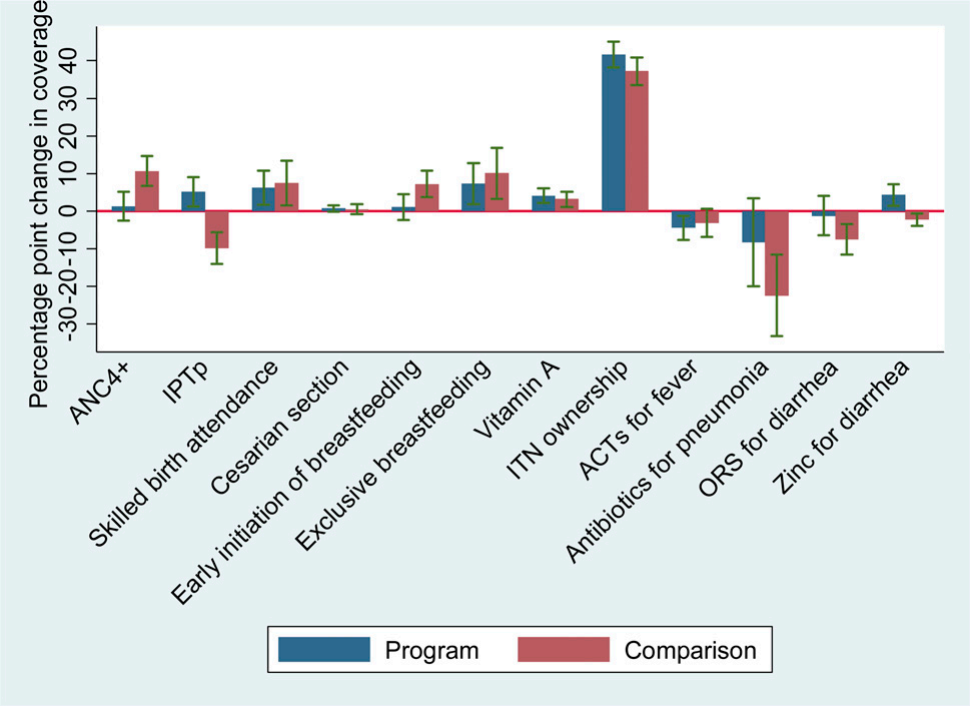
Changes in coverage in the program and comparison areas, from baseline to endline.

In program areas, we observed coverage increases from baseline to endline for a number of interventions targeted by the RSU in Burkina Faso (Figure 4

and Supplemental Web Annex 2). With the exception of ITN ownership, these coverage increases were relatively small (< 10 pp) and similar in magnitude to coverage increases in comparison areas. Coverage of key iCCM interventions, including antimalarials for fever, antibiotics for pneumonia, and ORS for diarrhea, decreased in program and comparison districts. We did observe a small but statistically significant increase in the treatment of diarrhea with zinc in the program area (from 4.0% [95% CI: 2.8–5.8] to 8.3% [95% CI: 6.2–11.0]) but not in the comparison area. Only two RSU targets for intervention coverage were achieved (intermittent preventive treatment of malaria in pregnancy and vitamin A supplementation) (Table 1). For four other interventions (skilled birth attendance, cesarean section, oral rehydration therapy [ORT] with continued feeding, and exclusive breast-feeding), the program targets, which were set based on data from 2003 and 2006, had already been reached at baseline.

The difference in differences analysis showed relatively small differences of differences between RSU and comparison areas (< 10 pp), with the exception of intermittent preventive treatment of malaria in pregnancy (IPTp) (14.9 pp [95% CI: 9.2–20.6], postpartum vitamin A (10.2 pp [95% CI: 4.7–15.8]), and antibiotics for pneumonia (14.2 pp [95% CI: −1.7 to 30.1]), all of which increased more (or decreased less) in RSU areas than in comparison areas (Table 5 and Supplemental Web Annex 2).

### Mortality changes.

**Figure 5.**
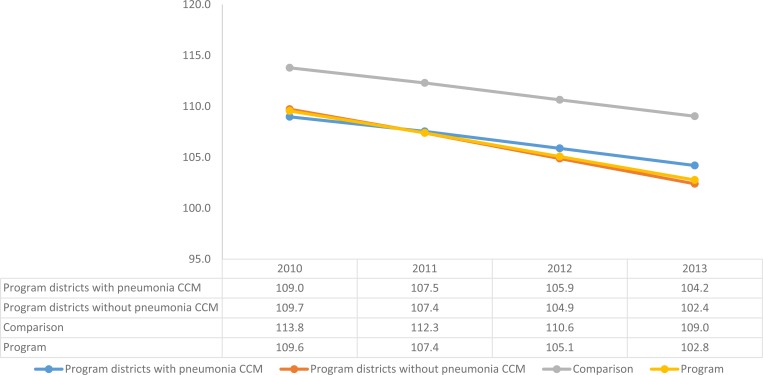
Modeled under-five mortality reductions in program and comparison areas, 2010–2013.

Projecting these coverage changes in LiST showed an estimated mortality reduction of 6.2% (range: 5–8.4%] in intervention areas (from 110 deaths per 1,000 live births to 103 [range: 100–104] per 1,000) versus 4.2% (range: 3.6–6.0%) in comparison areas (from 114 per 1,000 to 109 [range: 107–110] per 1,000) from 2010 to 2013 (Figure 5

). Mortality rates for mothers and newborns showed similar reductions in program and comparison areas, with the maternal mortality ratio declining by 2.5% [range: 1.9–3.5%] (from 416 per 100,000 to 405 [range: 402–408]) in program areas and 3.3% in comparison areas (from 434 per 100,000 to 419 [range: 415–423]), and neonatal mortality declining by 5.8% in program areas (from 26 per 1,000 to 24 [range: 24–25]) and 5.1% in comparison areas (from 27 per 1,000 to 26 [range: 26–26]).

Most of the estimated mortality reduction in both program and comparison areas was attributable to household ownership of ITNs. Increases in the coverage of this intervention accounted for 71% of the mortality reduction in program areas and 63% in comparison areas (Supplemental Web Annex 3, Figures 1.3 and 2.3).

### Contextual factors.

A quantitative description of key contextual variables is shown in Supplemental Web Annex 4, Table 1.4. The proportion of women of reproductive age with any formal education increased in both program and comparison areas from 2010 to 2013, and these increases were of similar magnitude (6.1 pp versus 5.1 pp). We observed significantly greater in-migration in comparison districts relative to program districts from 2010 to 2013 (comparison: 4.8% [95% CI: 4.2–5.3%]; RSU: 2.8% [95% CI: 2.5–3.3%]). Access to health facilities, as reported by the MoH,^20^,^21^ increased slightly in program areas while decreasing in comparison areas over the same time period.

In-depth interviews with district health personnel in program and comparison areas revealed similar trends in contextual factors that could have had an impact on MNCH over the program period. All districts reported poor harvests in 2011, but improved harvests and food security in 2012 and 2013. Gold mining activity and migration due to that activity was also a concern of health authorities across the program and comparison areas. Industrial gold mines are located in Kongoussi, Boulsa, and Ouahigouya districts in the program area and in Tenkodogo district in the comparison area, and smaller-scale “artisanal” gold mining is ongoing throughout the country, with the exception of the central region. Regional data on the number of gold mining sites in each region (province and district level data were not available) from 2010 to 2012 shows reductions in the overall number of gold mining sites in RSU regions during this period, but no overall change in the number of gold mining sites in regions containing comparison districts (Supplemental Web Annex 4, Table 2.4).^22^

A major contextual factor was the Global Fund-supported ITN distribution campaign and training of ASBCs to provide case management of malaria for children and adults. This program was implemented nationally, but the component providing CCM for malaria largely ended in 2012, and the endline coverage data show very low careseeking from ASBCs for children with fever in comparison areas (2.6%). No other major programs likely to have affected MNCH coverage indicators were identified in program or comparison areas.

## Discussion

This full prospective evaluation of the effectiveness of the RSU program in Burkina Faso included in-depth assessments of program implementation, quality of care, utilization, and intervention coverage changes, as well as modeled changes in mortality. We found important shortcomings in each of these areas and were unable to attribute any change in under-five, maternal, or neonatal mortality to the program. In this section, we discuss these findings and their implications as responses to a series of questions.

### To what extent was the program implemented? Were implementation, quality of care, and utilization sufficient to achieve population-level coverage changes?

Although initial iCCM implementation appeared to be strong, with over 3,000 ASBCs trained in 2010–2011, there were important shortcomings in both initial and ongoing implementation. In terms of the initial training, there were quality concerns including the use of nurses trained months before on iCCM to deliver the training, the failure to adapt training materials for illiterate ASBCs, the relatively large size of some training groups, and the lack of clinical practice for half of ASBCs. Thus, we would not expect this training to produce community workers able to provide high-quality care for sick children. In addition, in 2013, only 64% of the ASBCs who received initial iCCM training were providing iCCM services, suggesting an inefficient approach to training and deployment of ASBCs.

Ongoing iCCM implementation suffered from difficulties including infrequent supervision and lack of observation of case management during supervision. In addition, drug stockouts at the ASBC level were very common. Provision of clinical IMCI services suffered from some of the same difficulties, notably widespread stockouts of essential drugs and the fact that many first-level facilities did not have an IMCI-trained clinician present.

The implementation difficulties for both iCCM and clinical IMCI would be expected to lead to unsatisfactory quality of care for sick children, and this is what the evaluation found. All aspects of case management were poor at facility and community levels, with the exception of malaria case management in health facilities, which was quite good. Because the facility-level assessment was conducted in 2011, it is possible that facility-based care improved in program areas in 2012–2013. The community-based assessment, however, was conducted in 2013 and thus is a good representation of the quality of care provided by ASBCs at the end of the program.

Both quality of health services and utilization of these services must be high to produce population-level changes in coverage and mortality. We found that at community level, in addition to poor quality of care, utilization of ASBCs was very low. Low levels of utilization were likely attributable to the practice of charging for drugs, the fact that careseeking from facilities for sick children was relatively high at baseline, and the lack of a strong strategy to generate demand for iCCM. Given implementation challenges, poor quality of care at facility and community levels, and low utilization of ASBCs, there was no clear way for the RSU program to achieve significant changes in population-level coverage of interventions for sick children, and therefore few pathways to reducing under-five mortality, which was the program's overall objective.

### Were changes in coverage or mortality greater in the program area than in the comparison area?

Of the interventions targeted by the RSU program, there were only three for which coverage increased significantly more in the program area than in the comparison area: one antenatal (IPTp), one postnatal (postpartum vitamin A), and one for sick children (zinc treatment of diarrhea). Most program interventions, including zinc for diarrhea, saw only small coverage increases or even decreases from 2010 to 2013.

Because we modeled mortality change in program and comparison areas using LiST, a model in which mortality change is driven by intervention coverage change, it is not surprising that we saw small and similar levels of mortality reduction in both program and comparison areas. Without uncertainty bounds, we cannot determine whether the mortality reductions in program and comparison areas (−6.2% and −4.2%, respectively) were significantly different. However, we note that these reductions are qualitatively similar, that the sensitivity analysis produced overlapping ranges of possible mortality reductions in RSU and comparison areas, and that the reductions are very far from the 25% under-five mortality reduction targeted by the program.

### Could contextual factors have played a role in these findings?

Much of the estimated mortality reduction from LiST was attributable to increases in ITN coverage in program and comparison areas. Increases in ITN coverage were similar in program and comparison areas and were likely largely attributable to the Global Fund-supported ITN distribution campaigns. Although the RSU used program funds to pay for some of the ITNs distributed in the program areas, it seems likely that if these funds had not been available, an alternative source would have been found. Thus, our modeled mortality reductions are partially attributable to the Global Fund's activities.

We did not observe major differences in contextual factors between program and comparison areas that could have confounded the association (or lack thereof) between the program and intervention coverage. This does not exclude the possibility of unmeasured confounding, which is always a risk, particularly in nonrandomized evaluations. However, we believe that our findings with regard to intermediate factors (program implementation, quality of care, and utilization) sufficiently explain the coverage and mortality results. We note, however, that contextual factors, and particularly gold mining, may have acted as effect modifiers, in that they may have led to some attrition and dissatisfaction of ASBCs who may have seen gold mining as a more profitable occupation than community health work.^12^

### Implications for efforts to deliver iCCM at scale in Burkina Faso and similar contexts.

Based on the results of this evaluation, we cannot draw conclusions regarding whether iCCM as a strategy “works” in Burkina Faso, as the implementation of the strategy was flawed, perhaps because of pressure to implement quickly. Evaluators in other settings have also noted that attempts to rapidly scale up a complex intervention such as IMCI resulted in poor implementation and no impact.^35^ However, we can draw preliminary lessons regarding how to improve the implementation and results of such programs going forward. One important lesson is that when planning for training of health workers, quality matters. ASBC training was completed on a large scale and quite quickly, and, perhaps as a result, the quality of training appears to have been insufficient. A better approach might have been to scale up training more slowly, using trainers from the central MoH, small training groups, clinical practice, and training tools adapted for illiterate ASBCs.

A second lesson is the importance of the cadre delivering the program. Burkina Faso used an existing cadre of community health workers (ASBCs) to implement iCCM, and while their long-standing ties with their communities may have been an asset in program implementation, their low level of education and volunteer status may have impeded program success. Commendably, the MoH and partners have developed a new policy on ASBCs^13^ that aims to address many of the issues identified in the evaluation, although it is not yet clear how this policy will be operationalized.

A final lesson is the importance of taking into account drug supply systems and demand generation in the design of an iCCM program. Widespread stockouts at community level made it difficult for ASBCs to provide adequate case management, and the low level of utilization of ASBCs likely limited the impact of the iCCM program. In addition, stockouts were not systematically monitored by the program and therefore were not identified as a problem until the Independent Evaluation Team's survey of ASBCs in 2013.

The findings regarding utilization and careseeking point to the need for programs to develop, before implementation, a clear strategy regarding the population to be targeted by a particular program, and regarding how to increase demand in the target population. An equity analysis to assess the types of households that sought care from different sources is underway and will be published separately. This analysis may provide the basis for recommendations on how to reach those sick children who are taken neither to a health facility nor to an ASBC.

### Limitations.

This evaluation had a number of limitations. Due to delays in inviting the evaluation team to Burkina Faso and initial funding delays, the baseline survey was conducted in 2010, although some health facility activities had started in 2009. While this should not affect our assessment of the impact of iCCM, it means that we may not have captured the full effect of facility-based activities. There were also timing issues with respect to the endline survey: Because the transfer of funds for the survey was delayed by the primary grantee, the endline was conducted in a slightly different season than the baseline (November–March versus August–January). A previous study on seasonal differences in careseeking in Burkina Faso,^36^ as well as a reanalysis of 2010 Demographic and Health Survey data by season (Supplemental Web Annex 5, Tables 1.15 and 2.5), suggests that this difference should have favored the program (i.e., careseeking for childhood illness is expected to be higher after the rainy season than during the rainy season). In addition, we would expect the seasonal effect to be similar in both intervention and comparison areas. However, we cannot exclude the possibility that this seasonal difference in baseline and endline surveys affected our difference in differences analysis.

The evaluation included one round of facility-level quality of care assessments and one round of community-level implementation and quality of care assessments. To better capture changes in program implementation and quality of care, it would have been preferable to conduct two rounds of assessments and to conduct the facility assessments in both program and comparison areas. An economic assessment was also planned as a part of the original design, but could not be completed because the facility quality of care assessment was conducted only in the program areas, and there was therefore no basis for cost comparisons.

This evaluation focused primarily on assessing whether the program achieved its targets, which were formulated in terms of intervention coverage and under-five mortality. We did not conduct an in-depth assessment of policy inputs and effects of the program, although we tried to document these as we learned about them through meetings with implementing partners.

One of the RSU program indicators was the proportion of children with suspected pneumonia who received antibiotics. Validation studies published in 2013^37^ concluded that this indicator has poor validity when measured in household surveys, as respondents cannot report accurately on the numerator or denominator. The evaluation still measured this indicator and observed a large decrease, but we put little weight on that decrease because of the concerns regarding the validity of the indicator. In addition to this indicator, we also reported on careseeking for children with symptoms of pneumonia, which has fewer validity concerns (and which also decreased over the program period).

Finally, this evaluation used a nonrandomized design by necessity, as the program districts had already been selected by the time the evaluation team was invited to Burkina Faso and randomization was not an option. We instead used a group-matched design and found that our program and comparison areas were very similar at baseline. We also attempted to measure contextual factors to account for potential confounding. However, unmeasured confounding cannot be ruled out with this type of design.

## Conclusions

Although the RSU did result in a number of policy changes at program outset and later on in response to evaluation findings, implementation of the RSU program itself and in particular the iCCM component was not strong enough to achieve population-level changes in coverage or mortality. Encouragingly, the Burkina Faso MoH and partners have indicated that they will incorporate evaluation findings in future MNCH programming. These results should not be interpreted as evidence that iCCM cannot result in coverage or mortality changes, but rather that it did not result in changes in this setting given weak implementation, poor quality of care, and low utilization. It is notable, however, that very few settings have been able to implement CCM at scale with strong enough intensity and quality to achieve mortality and coverage impacts.^38^ More attention to the implementation and quality of these programs is needed, with perhaps a longer timeframe for design and scale-up to ensure that implementation challenges are adequately addressed.

## Supplementary Material

Supplemental Datas.

## Figures and Tables

**Table 1:** Program targets and baseline and endline levels of mortality and coverage

Indicator	Target	Baseline (2010)	Endline (2013)
Under-five mortality rate	25% reduction from baseline to endline (82.5 deaths per 1,000)	110 deaths per 1,000	103 deaths per 1,000
Antenatal care - 4 or more visits (ANC4+)	80%	44%	45%
IPTp	70%	39%	44%
Skilled birth attendance	60%	73%	80%
Cesarean section	2%	2%	3%
Early initiation of breast-feeding	40%	25%	26%
Postpartum vitamin A	60%	50%	57%
ACT for fever	70%	27%	23%
Antibiotics for pneumonia	60%	30%	16%
ORT + continued feeding	60%	65%	64%
ITNs	70%	51%	92%
Exclusive breast-feeding	20%	35%	42%
Vitamin A supplementation	90%	89%	93%

ACT = artemisinin combination therapy; IPTp = intermittent preventive treatment of malaria in pregnancy; ITNs = insecticide-treated bednets; ORT = oral rehydration therapy.

**Table 2:** Implementation and quality of iCCM in program areas, 2013

	*n*/*N*	%	95% CI
Implementation			
ASBCs providing iCCM services who received CCM training	380/385	98.6	95.8–99.5
ASBCs providing iCCM services who received CCM training with clinical practice	201/385	52.2	45.9–58.4
ASBCs providing iCCM services who received at least one CCM supervision	304/385	78.9	72.7–84.0
Time since last supervision			
0–2 months	147/385	38.1	32.3–44.2
3+ months/never supervised	239/385	61.9	55.8–67.8
Activities during supervision			
Supervisor observed sick child consultation	90/304	29.6	23.3–36.9
Supervisor gave case scenarios	46/304	15.3	11.2–20.5
ASBCs providing iCCM services who received initial CCM drug kit	380/385	98.8	95.4–99.7
ASBCs providing iCCM services who had all essential drugs in stock on the day of the visit	34/385	8.8	6.0–12.7
Quality of care			
Child assessed for four danger signs	78/724	11	8.2–14.1
Child checked for cough/difficult breathing, diarrhea, and fever	248/724	34	29.0–40.1
Child correctly classified for diarrhea and fever (and for pneumonia in pneumonia CCM districts)*	472/718	66	60.5–70.5
Child with uncomplicated illness correctly managed for diarrhea and fever (and for pneumonia in pneumonia CCM districts)*	240/668	36	31.3–40.9
Child not needing an antibiotic did not receive an antibiotic (in pneumonia CCM districts)*	58/72	81	74.7–85.2
Child needing referral was referred	51/177	29	20.0–939.0

ASBCs = Agents de santé à base communautaires; CI = confidence interval; iCCM = integrated community case management.

* Excludes six children with danger signs who were immediately referred.

**Table 3:** Quality of care for sick children in health facilities in program areas, 2011

	*n*/*N*	%
Child assessed for three danger signs	70/250	28.0
Child checked for cough/difficult breathing, diarrhea, and fever	206/250	82.4
Children for whom health worker's classification matches gold standard classification	21/136	15.4
Children with uncomplicated malaria for whom ACTs were correctly prescribed	168/185	90.8
Children with pneumonia for whom antibiotics were correctly prescribed	13/38	34.2
Children with diarrhea for whom ORS and zinc were correctly prescribed	21/116	18.1
Children with diarrhea for whom ORS was correctly prescribed	35/116	30.2
Children needing referral were referred	8/20	40.0

ACTs = artemisinin combination therapies; ORS = oral rehydration solution.

**Table 4:** Careseeking in program and comparison districts, at baseline (August 2010 to January 2011) and endline (November 2013 to March 2014)

	ASBC				Facility			
	Baseline		Endline		Baseline		Endline	
	%	95% CI	%	95% CI	%	95% CI	%	95% CI
Careseeking for fever, diarrhea, or suspected pneumonia								
Program	4.7	3.7–6.0	6.7	5.5–8.2	56.5	53.6–59.3	47.2	44.3–50.3
Comparison	1.9	1.4–2.7	2.3	1.4–3.7	50.6	47.3–53.9	43.4	40.1–46.7
Careseeking for fever								
Program	4.5	3.5–5.8	7.2	5.7–8.9	58.4	55.5–61.2	48.7	45.4–52.0
Comparison	2.1	1.5–3.0	2.6	1.6–4.2	53.5	50.1–56.8	45.8	42.1–49.5
Careseeking for suspected pneumonia								
Program districts with pneumonia CCM	5.5	2.0–14.5	10.2	6.0–16.7	55.7	45.6–65.3	52.1	44.1–59.9
Comparison	0.7	0.1–4.6	1.9	0.6–5.3	62.1	52.6–70.7	54.7	46.9–61.4
Careseeking for diarrhea								
Program	3.5	2.3–5.1	4.2	3.0–6.0	43.1	39.3–47.0	44.3	40.4–48.2
Comparison	0.6	0.2–1.6	0.5	0.2–1.4	33.2	29.0–37.7	30.7	27.3–34.3

ASBC = Agents de santé à base communautaire; CCM = community case management; CI = confidence interval.

**Table 5:** Difference in differences analysis for coverage of key program interventions

Intervention	Difference in differences in intervention coverage between program and comparison areas, 2010–2013	
	Percentage points	95% CI
Antenatal care - 4 or more visits (ANC4+)	−9.3†	−14.8 to −3.8
IPTp	14.9‡	9.2 to 20.6
Skilled birth attendance	−1.2	−8.7 to 6.3
Cesarean section	0.2	−1.4 to 1.8
Postpartum vitamin A	10.2‡	4.7 to 15.8
Early breast-feeding initiation	−6.2	−11.1 to 1.2
Exclusive breast-feeding	−2.6	−11.4 to 6.1
Vitamin A supplementation	0.9	−2.0 to 3.8
ACTs for malaria	−1.4	−6.2 to 3.5
Antibiotics for pneumonia*	14.2	−1.7 to 30.1
ORS for diarrhea	6.2	−0.3 to 12.8
Zinc for diarrhea	6.6‡	3.2 to 9.9
ITN ownership	4.4	−0.6 to 9.5

ACTs = artemisinin combination therapies; CI = confidence interval; IPTp = intermittent preventive treatment of malaria in pregnancy; ITN = insecticide-treated bednet; ORS = oral rehydration solution.

* Program districts with pneumonia CCM relative to comparison districts.

† Coverage increased significantly more in comparison areas relative to program areas, *P* < 0.05.

‡ Coverage increased significantly more in program areas relative to comparison areas, *P* < 0.05.
