# Supplementary material for: Independent Evaluation of the Rapid Scale-Up Program to Reduce Under-Five Mortality in Burkina Faso
Source: Am J Trop Med Hyg. 2016 Mar 2;94(3):584–95. doi: 10.4269/ajtmh.15-0585 (PMC4775895; doi:10.4269/ajtmh.15-0585)
Supplement: Supplementary file 1 [file SD7.pdf]

SUPPLEMENTAL WEB ANNEX 1: CHARACTERISTICS OF THE EVALUATION DISTRICTS

Supplemental Table 1.1
Indicators used to match comparison districts to program districts

	% Rural population who are poor (2006 census)	First level facility catchment radius (km) (2008 Health Statistics Yearbook)	% Population ≥ 25 years old who never attended school (2006 census)	Number of first level facilities with minimum requisite staff per 10,000 pop (2008 Health Statistics Yearbook)	Number of nurses per 10,000 pop (2008 Health Statistics Yearbook)	Population (2008 Health Statistics Yearbook)
Program districts						
Barsalogho	49.5%	9.51	91.5%	0.70	2.44	184,452
Boulsa	63.5%	9.11	93.7%	0.70	2.33	330,452
Gourcy	48.6%	7.29	90.8%	0.84	2.77	165,927
Kaya	49.5%	5.52	91.5%	0.60	1.34	484,932
Kongoussi	52.2%	7.46	88.7%	0.84	3.42	263,408
Ouahigouya	49.7%	3.19	86.7%	1.08	2.33	399,474
Seguenega	49.7%	8.93	86.7%	0.82	3.23	158,120
Titao	64.3%	8.27	91.4%	0.66	2.11	166,113
Yako	40.6%	3.51	90.4%	0.36	2.24	356,554
Average	52.0%	6.98	90.1%	0.73	2.47	278,826
Comparison districts						
Tenkodogo	35.8%	5.37	89.3%	0.71	2.13	183,527
Bousse	43.3%	4.91	92.0%	1.13	2.48	132,820
Koudougou	25.6%	4.22	84.9%	0.72	3.20	415,303
Sapouy	60.6%	9.94	90.9%	0.85	1.93	176,068
Po	49.8%	7.46	86.8%	1.01	4.26	148,006
Bogande	73.0%	9.66	94.1%	0.30	1.79	328,725
Fada	67.0%	9.22	88.5%	0.69	1.88	319,882
Average	50.7%	7.25	89.5%	0.77	2.52	243,476

Supplemental Table 2.1
Key baseline characteristics for intervention and comparison areas

	Rapid scale-up districts			
	All districts	Pneumonia CCM districts	Non-pneumonia CCM districts	Comparison districts
ITN ownership (% households that own one or more ITN)	50.6% (47.7–53.6)	50.0% (46.7–53.2)	50.7% (47.3–54.1)	52.9% (49.6–56.2)
ANC 4+ (% women who attended at least 4 ANC consultations during pregnancy in last 2 years)	43.6% (41.0–46.3)	38.2% (35.0–41.3)	44.6% (41.5–47.6)	47.5% (44.9–50.1)
Skilled birth attendance (% live births delivered by a skilled attendant in last 2 years)	73.3% (70.1–76.5)	74.1% (69.7–78.6)	73.1% (69.4–76.8)	68.1% (64.0–72.1)
Children completely vaccinated (% children 12-23 months who have received all recommended vaccines)	85.1% (82.9–87.3)	88.0% (85.2–90.8)	84.6% (82.0–87.2)	80.8% (77.6–84.0)
ACTs for fever (% children with fever in last 2 weeks who received an ACT)	27.1% (24.9–29.4)	30.5% (26.9–34.1)	26.5% (24.0–29.0)	25.2% (22.9–27.6)
Antibiotics for suspected pneumonia (% children with suspected pneumonia in last 2 weeks who received antibiotics)	29.8% (24.2–36.2)	32.3% (23.2–42.8)	29.5% (23.4–36.6)	39.1% (30.9–48.0)
ORS for diarrhea (% children with diarrhea in last 2 weeks who received ORS)	26.5% (23.2–30.0)	14.0% (11.2–17.3)	28.8% (24.9–32.9)	17.6% (14.6–20.9)
Zinc for diarrhea (% children with diarrhea in last 2 weeks who received zinc)	4.0% (2.8–5.8)	2.0% (1.1–3.8)	4.4% (2.9–6.5)	3.4% (2.2–5.3)

SUPPLEMENTAL WEB ANNEX 2: COVERAGE RESULTS FROM BASELINE AND ENDLINE SURVEYS

Supplemental Table 1.2

ANC coverage among women with a live birth in the 24 months prior to the survey, at baseline (2010) and endline (2013)

	Women with a live birth last 24 months having received at least 4 ANC visits							
	Baseline			Endline			Change	
	N	%	95% CI	N	%	95% CI	pp	95% CI
Program area	4,815	43.6	[41.0–46.3]	4,959	45.0	[42.2–47.8]	1.3	[−2.5, 5.2]
Districts with pneumonia CCM	714	38.2	[35.0–41.3]	725	43.9	[41.0–46.8]	**5.7***	[1.3, 10.0]
Districts without pneumonia CCM	4,101	44.6	[41.5–47.6]	4,233	45.2	[41.9–48.4]	0.6	[−3.9, 5.0]
Comparison area	3,063	47.5	[44.9–50.1]	2,903	58.1	[55.2–61.0]	**10.6***	[6.7, 14.6]
Difference of differences – Program versus comparison area	–	–	–	–	–	–	**−9.3***	[−14.8, −3.8]

*Bolded values indicate a statistically significant difference ($P < 0.05$).

Supplemental Table 2.2

Coverage of IPTp among women with a live birth in the 24 months prior to the survey, at baseline (2010) and endline (2013)

	Women with a live birth last 24 months who received at least 2 doses of sulfadoxine-pyrimethamine during the pregnancy							
	Baseline			Endline			Change	
	N	%	95% CI	N	%	95% CI	pp	95% CI
Program area	4,815	39.2	[36.4–42.0]	4,920	44.3	[41.4–47.2]	**5.1***	[1.3, 9.0]
Districts with pneumonia CCM	714	35.8	[32.8–38.8]	721	46.7	[43.3–50.1]	**10.9***	[6.2, 15.5]
Districts without pneumonia CCM	4,101	39.8	[36.6–43.0]	4,199	43.9	[40.6–47.2]	4.1	[−0.3, 8.6]
Comparison area	3,063	36.3	[33.2–39.4]	2,888	26.5	[23.6–29.4]	**−9.8***	[−14.0, −5.6]
Difference of differences – Program versus comparison area	–	–	–	–	–	–	**14.9***	[9.2, 20.6]

*Bolded values indicate a statistically significant change ($P < 0.05$) from baseline to endline.

Supplemental Table 3.2

Coverage of skilled birth attendance among women with a live birth in the 24 months prior to the survey, at baseline (2010) and endline (2013)

	Women with a live birth last 24 months assisted by a skilled attendant at birth							
	Baseline			Endline			Change	
	N	%	95% CI	N	%	95% CI	pp	95% CI
Program area	4,815	73.3	[70.1–76.5]	4,959	79.5	[76.6–82.4]	**6.2***	[1.7, 10.7]
Districts with pneumonia CCM	714	74.1	[69.7–78.6]	725	71.6	[67.6–75.5]	−2.6	[−8.6, 3.5]
Districts without pneumonia CCM	4,101	73.1	[69.4–76.8]	4,233	80.9	[77.5–84.2]	**7.7***	[2.5, 12.9]
Comparison area	3,063	68.1	[64.0–72.1]	2,904	75.5	[71.6–79.4]	**7.4***	[1.5, 13.4]
Difference of differences – Program versus comparison area	–	–	–	–	–	–	−1.2	[−8.7, 6.3]

*Bolded values indicate a statistically significant change ($P < 0.05$) from baseline to endline.

Supplemental Table 4.2

Coverage of cesarean section among women with a live birth in the 24 months prior to the survey, at baseline (2010) and endline (2013)

	Women with a live birth last 24 months who received a cesarean section							
	Baseline			Endline			Change	
	N	%	95% CI	N	%	95% CI	pp	95% CI
Program area	4,815	1.9	[1.3–2.4]	4,959	2.6	[2.0–3.2]	0.8	[−0.1, 1.6]
Districts with pneumonia CCM	714	1.6	[1.0–2.3]	725	2.7	[1.8–3.6]	1.1	[0.0, 2.2]
Districts without pneumonia CCM	4,101	1.9	[1.3–2.5]	4,233	2.6	[1.9–3.3]	0.7	[−0.2, 1.6]
Comparison area	3,063	3.3	[2.5–4.1]	2,904	3.8	[2.7–5.0]	0.5	[−0.8, 1.9]
Difference of differences – Program versus comparison area	–	–	–	–	–	–	0.2	[−1.4, 1.8]

Supplemental Table 5.2

Coverage of early initiation of breastfeeding among women with a live birth in the 24 months prior to the survey, at baseline (2010) and endline (2013)

	Women with a live birth last 24 months who breastfed the newborn within 1 hour of birth							
	Baseline			Endline			Difference	
	N	%	95% CI	N	%	95% CI	pp	95% CI
Program area	4,815	25.3	[22.7–28.0]	4,959	26.4	[24.0–28.7]	1.0	[−2.4, 4.5]
Districts with pneumonia CCM	714	28.7	[25.4–32.1]	725	22.9	[20.7–25.1]	**−5.9***	[−9.6, −2.1]
Districts without pneumonia CCM	4,101	24.7	[21.7–27.8]	4,233	27.0	[24.2–29.7]	2.2	[−1.7, 6.2]
Comparison area	3,063	23.7	[21.4–26.1]	2,904	30.9	[28.1–33.7]	**7.2***	[3.7, 10.7]
Difference of differences – Program versus comparison area	–	–	–	–	–	–	−6.2	[−11.1, 1.2]

*Bolded values indicate a statistically significant change ($P < 0.05$) from baseline to endline.

Supplemental Table 6.2

Coverage of postpartum vitamin A among women with a live birth in the 24 months prior to the survey, at baseline (2010) and endline (2013)

	Women who received a dose of vitamin A within 8 weeks of birth							
	Baseline			Endline			Difference	
	N	%	95% CI	N	%	95% CI	pp	95% CI
Program area	4,815	49.7	[47.1–52.3]	4,959	57.3	[54.6–60.0]	**7.6***	[3.8, 11.4]
Districts with pneumonia CCM	714	53.5	[49.8–57.1]	725	51.8	[49.1–54.4]	−1.7	[−6.2, 2.8]
Districts without pneumonia CCM	4,101	49.0	[46.0–52.0]	4,233	58.2	[55.1–61.3]	**9.2***	[4.8, 13.6]
Comparison area	3,063	41.7	[38.9–44.5]	2,904	39.0	[36.1–42.0]	−2.6	[−6.7, 14.1]
Difference of differences – Program versus comparison area	–	–	–	–	–	–	**10.2***	[4.7, 15.8]

*Bolded values indicate a statistically significant change ($P < 0.05$) from baseline to endline.

Supplemental Table 7.2

Proportion of children aged 0–5 months exclusively breastfed, at baseline (2010) and endline (2013)

	Children 0–5 months exclusively breastfed							
	Baseline			Endline			Difference	
	N	%	95% CI	N	%	95% CI	pp	95% CI
Program area	1,385	34.5	[31.2–37.8]	1,285	41.8	[37.6–46.1]	**7.3***	[1.9, 12.8]
Districts with pneumonia CCM	206	35.4	[30.7–40.0]	181	40.6	[36.4–44.7]	5.2	[−1.0, 11.4]
Districts without pneumonia CCM	1,179	34.3	[30.5–38.1]	1,104	42.0	[37.1–46.9]	**7.7***	[1.4, 13.9]
Comparison area	867	30.5	[26.4–34.6]	653	40.5	[35.4–45.5]	**10.1***	[3.2, 16.8]
Difference of differences – Program versus comparison area	–	–	–	–	–	–	−2.6	[−11.4, 6.1]

*Bolded values indicate a statistically significant change ($P < 0.05$) from baseline to endline.

Supplemental Table 8.2

Coverage of vitamin A supplementation among children 6–59 months, at baseline (2010) and endline (2013)

	Children 6–59 months who received a dose of vitamin A in the last 6 months							
	Baseline			Endline			Difference	
	N	%	95% CI	N	%	95% CI	pp	95% CI
Program area	9,789	88.9	[87.3–90.4]	11,042	92.9	[91.8–94.1]	**4.1***	[2.1, 6.1]
Districts with pneumonia CCM	1,380	93.5	[92.0–95.0]	1,553	92.6	[91.5–93.6]	−0.9	[−2.7, 0.9]
Districts without pneumonia CCM	8,409	88.1	[86.4–89.8]	9,488	93.0	[91.6–94.4]	**4.9***	[2.6, 7.2]
Comparison area	5,980	89.0	[87.4–90.7]	6,392	92.2	[90.9–93.5]	**3.2***	[1.1, 5.2]
Difference of differences – Program versus comparison area	–	–	–	–	–	–	0.9	[−2.0, 3.8]

*Bolded values indicate a statistically significant change ($P < 0.05$) from baseline to endline.

Supplemental Table 9.2

Coverage of careseeking from a health facility among children < 5 years with fever, at baseline (2010) and endline (2013)

	Children < 5 years with fever in the last 2 weeks who were taken to a health facility							
	Baseline			Endline			Difference	
	N	%	95% CI	N	%	95% CI	pp	95% CI
Program area	3,639	58.4	[55.5–61.2]	3,057	48.7	[45.4–52.0]	**−9.7***	[−14.1, −5.2]
Districts with pneumonia CCM	563	56.1	[52.4–59.8]	410	40.8	[37.5–44.2]	**−15.3***	[−20.8, −9.7]
Districts without pneumonia CCM	3,076	58.8	[55.4–62.0]	2,646	50.0	[46.2–53.7]	**−8.8***	[−13.9, −3.7]
Comparison area	2,338	53.5	[50.1–56.8]	2,178	45.8	[42.1–49.5]	**−7.7***	[−12.9, −2.5]
Difference of differences – Program versus comparison area	–	–	–	–	–	–	−1.9	[−8.8, 4.9]

*Bolded values indicate a statistically significant change ($P < 0.05$) from baseline to endline.

Supplemental Table 10.2

Coverage of careseeking from an ASBC among children < 5 years with fever, at baseline (2010) and endline (2013)

	Children < 5 years with fever in the last 2 weeks who were taken to an ASBC							
	Baseline			Endline			Difference	
	N	%	95% CI	N	%	95% CI	pp	95% CI
Program area	3,639	4.5	[3.5–5.8]	3,057	7.2	[5.7–8.9]	**2.6***	[0.7, 4.6]
Districts with pneumonia CCM	563	2.6	[1.8–3.7]	410	9.4	[7.6–11.5]	**6.8***	[4.6, 9.0]
Districts without pneumonia CCM	3,076	4.9	[3.7–6.4]	2,646	6.8	[5.2–8.8]	1.9	[−0.2, 4.2]
Comparison area	2,338	2.1	[1.5–3.0]	2,178	2.6	[1.6–4.2]	0.5	[−1.0, 2.0]
Difference of differences – Program versus comparison area	–	–	–	–	–	–	2.1	[−0.3, 4.6]

*Bolded values indicate a statistically significant change ($P < 0.05$) from baseline to endline.

Supplemental Table 11.2

Coverage of ACTs among children < 5 years with fever, at baseline (2010) and endline (2013)

	Children < 5 years with fever in the last 2 weeks who received an ACT							
	Baseline			Endline			Difference	
	N	%	95% CI	N	%	95% CI	pp	95% CI
Program area	3,639	27.1	[24.9–29.4]	3,057	22.7	[20.5–24.8]	**−4.4***	[−7.6, −1.3]
Districts with pneumonia CCM	563	30.5	[26.9–34.1]	410	21.9	[19.7–24.2]	**−8.6***	[−13.0, −4.1]
Districts without pneumonia CCM	3,076	26.5	[24.0–29.0]	2,646	22.8	[20.5–25.3]	**−3.7***	[−7.3, −0.1]
Comparison area	2,338	25.2	[22.9–27.6]	2,178	22.2	[19.4–24.9]	−3.1	[−6.8, 0.6]
Difference of differences – Program versus comparison area	–	–	–	–	–	–	−1.4	[−6.2, 3.5]

*Bolded values indicate a statistically significant change ($P < 0.05$) from baseline to endline.

Supplemental Table 12.2

Coverage of careseeking from a health facility among children < 5 years with suspected pneumonia, at baseline (2010) and endline (2013)

	Children < 5 years with suspected pneumonia in the last 2 weeks who were taken to a health facility							
	Baseline			Endline			Difference	
	N	%	95% CI	N	%	95% CI	pp	95% CI
Program area	307	62.7	[55.1–69.8]	530	54.4	[48.3–60.4]	−8.3	[−17.9, 1.3] ($P = 0.09$)
Districts with pneumonia CCM	32	55.7	[45.6–65.3]	58	52.1	[44.1–59.9]	−3.6	[−16.5, 9.3]
Districts without pneumonia CCM	275	63.6	[55.0–71.3]	472	54.7	[47.9–61.4]	−8.9	[−19.5, 1.8]
Comparison area	164	62.1	[52.6–70.7]	220	54.2	[46.9–61.4]	−7.9	[−19.7, 3.9]
Difference of differences – Program versus comparison area	–	–	–	–	–	–	4.3	[−13.2, 21.7]

Supplemental Table 13.2

Coverage of careseeking from an ASBC among children < 5 years with suspected pneumonia, at baseline (2010) and endline (2013)

	Children < 5 years with suspected pneumonia in the past 2 weeks who were taken to an ASBC							
	Baseline			Endline			Difference	
	N	%	95% CI	N	%	95% CI	pp	95% CI
Program area	307	5.2	[2.6–10.4]	530	5.2	[3.3–8.2]	0.0	[−4.4, 4.4]
Districts with pneumonia CCM	32	5.5	[2.0–14.5]	58	10.2	[6.0–16.7]	4.6	[−3.0, 12.3]
Districts without pneumonia CCM	275	5.2	[2.3–11.1]	472	4.6	[2.6–8.1]	−0.6	[−5.4, 4.2]
Comparison area	164	0.7	[0.1–4.6]	220	1.9	[0.6–5.3]	1.2	[−1.2, 3.6]
Difference of differences – Program versus comparison area	–	–	–	–	–	–	3.4	[−4.6, 11.4]

Supplemental Table 14.2

Coverage of antibiotics among children < 5 years with suspected pneumonia, at baseline (2010) and endline (2013)

	Children < 5 years with suspected pneumonia in the past 2 weeks who received an antibiotic							
	Baseline			Endline			Difference	
	N	%	95% CI	N	%	95% CI	pp	95% CI
Program area	307	29.8	[24.2–36.2]	530	16.1	[12.4–20.7]	**−13.7***	[−21.1, −6.3]
Districts with pneumonia CCM	32	32.3	[23.2–42.8]	58	24.0	[18.3–30.7]	−8.3	[−19.9, 3.4]
Districts without pneumonia CCM	275	29.5	[23.4–36.6]	472	15.2	[11.1–20.3]	**−14.3***	[−22.5, −6.2]
Comparison area	164	39.1	[30.9–48.0]	220	16.6	[11.0–24.4]	**−22.5***	[−33.3, −11.6]
Difference of differences – Program versus comparison area	–	–	–	–	–	–	14.2	[−1.7, 30.1]

*Bolded values indicate a statistically significant change ($P < 0.05$) from baseline to endline.

Supplemental Table 15.2

Coverage of careseeking from a health facility among children < 5 years with diarrhea, at baseline (2010) and endline (2013)

	Children < 5 years with diarrhea in the past 2 weeks who were taken to a health facility							
	Baseline			Endline			Difference	
	N	%	95% CI	N	%	95% CI	pp	95% CI
Program area	1,431	43.1	[39.3–47.0]	1,627	44.3	[40.4–48.2]	1.2	[−4.6, 6.9]
Districts with pneumonia CCM	223	41.9	[37.4–46.6]	218	33.1	[29.6–36.9]	**−8.8***	[−15.0, −2.6]
Districts without pneumonia CCM	1,208	43.4	[38.9–47.9]	1,409	46.0	[41.6–50.5]	2.6	[−3.9, 9.3]
Comparison area	715	33.2	[29.0–37.7]	1,014	30.7	[27.3–34.3]	−2.5	[−8.2, 3.1]
Difference of differences – Program versus comparison area	–	–	–	–	–	–	3.7	[−4.4, 11.8]

*Bolded values indicate a statistically significant change ($P < 0.05$) from baseline to endline.

Supplemental Table 16.2

Coverage of careseeking from an ASBC among children < 5 years with diarrhea, at baseline (2010) and endline (2013)

	Children < 5 years with diarrhea in the past 2 weeks who were taken to an ASBC							
	Baseline			Endline			Difference	
	N	%	95% CI	N	%	95% CI	pp	95% CI
Program area	1,431	3.5	[2.3–5.1]	1,627	4.2	[3.0–6.0]	0.8	[−1.2, 2.8]
Districts with pneumonia CCM	223	2.3	[1.4–3.9]	218	8.3	[6.4–10.8]	**6.0***	[3.5, 8.5]
Districts without pneumonia CCM	1,208	3.7	[2.4–5.6]	1,409	3.6	[2.2–5.7]	−0.1	[−2.4, 2.2]
Comparison area	715	0.6	[0.2–1.6]	1,014	0.5	[0.2–1.4]	−0.1	[−0.9, 0.7]
Difference of differences – Program versus comparison area	–	–	–	–	–	–	0.8	[−1.3, 3.0]

*Bolded values indicate a statistically significant change ($P < 0.05$) from baseline to endline.

Supplemental Table 17.2

Coverage of ORS among children < 5 years with diarrhea, at baseline (2010) and endline (2013)

	Children < 5 years with diarrhea in the past 2 weeks who received ORS							
	Baseline			Endline			Difference	
	N	%	95% CI	N	%	95% CI	pp	95% CI
Program area	1,431	26.5	[23.2–30.0]	1,627	25.2	[22.1–28.7]	−1.2	[−6.4, 4.0]
Districts with pneumonia CCM	223	14.0	[11.2–17.3]	218	16.6	[14.2–19.4]	2.6	[−1.4, 6.6]
Districts without pneumonia CCM	1,208	28.8	[24.9–32.9]	1,409	26.6	[23.0–30.5]	−2.2	[−8.2, 3.8]
Comparison area	715	17.6	[14.6–20.9]	1,014	10.1	[8.0–12.8]	**−7.5***	[−11.5, −3.5]
Difference of differences – Program versus comparison area	–	–	–	–	–	–	6.2	[−0.3, 12.8]

*Bolded values indicate a statistically significant change ($P < 0.05$) from baseline to endline.

Supplemental Table 18.2

Coverage of zinc among children < 5 years with diarrhea, at baseline (2010) and endline (2013)

	Children < 5 years with diarrhea in the past 2 weeks who received zinc							
	Baseline			Endline			Difference	
	N	%	95% CI	N	%	95% CI	pp	95% CI
Program area	1,431	4.0	[2.8–5.8]	1,627	8.3	[6.2–11.0]	**4.3***	[1.4, 7.2]
Districts with pneumonia CCM	223	2.0	[1.1–3.8]	218	9.0	[6.9–11.5]	**7.0***	[4.3, 9.5]
Districts without pneumonia CCM	1,208	4.4	[2.9–6.5]	1,409	8.2	[5.9–11.4]	**3.8***	[0.5, 7.2]
Comparison area	715	3.4	[2.2–5.3]	1,014	1.2	[0.7–2.1]	**−2.2***	[−3.9, −0.6]
Difference of differences – Program versus comparison area	–	–	–	–	–	–	**6.6***	[3.2, 9.9]

*Bolded values indicate a statistically significant change ($P < 0.05$) from baseline to endline.

Supplemental Table 19.2

Proportion of households that own one or more ITN, at baseline (2010) and endline (2013)

	Households owning an ITN							
	Baseline			Endline			Difference	
	N	%	95% CI	N	%	95% CI	pp	95% CI
Program area	10,548	50.6	[47.7–53.6]	10,330	92.2	[90.7–93.8]	**41.6***	[38.2, 45.0]
Districts with pneumonia CCM	1,333	50.0	[46.7–53.2]	1,306	96.8	[96.0–97.5]	**46.8***	[43.5, 50.1]
Districts without pneumonia CCM	9,215	50.7	[47.3–54.1]	9,024	91.6	[89.8–93.3]	**40.8***	[36.9, 44.7]
Comparison area	6,591	52.9	[49.6–56.2]	6,455	90.0	[88.3–91.8]	**37.2***	[33.5, 40.8]
Difference of differences – Program versus comparison area	–	–	–	–	–	–	4.4	[−0.6, 9.5]

*Bolded values indicate a statistically significant change ($P < 0.05$) from baseline to endline.

SUPPLEMENTAL WEB ANNEX 3: UNDER-FIVE MORTALITY REDUCTION BY INTERVENTION

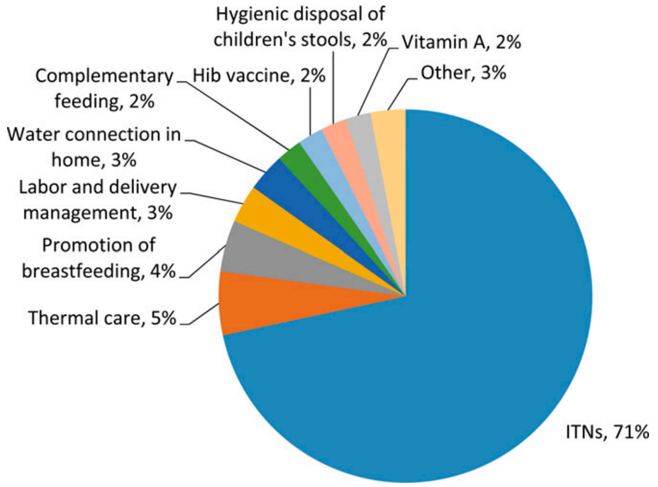

Supplemental Figure 1. Percentage of under-five mortality reduction in the program area by intervention, 2010–2013.

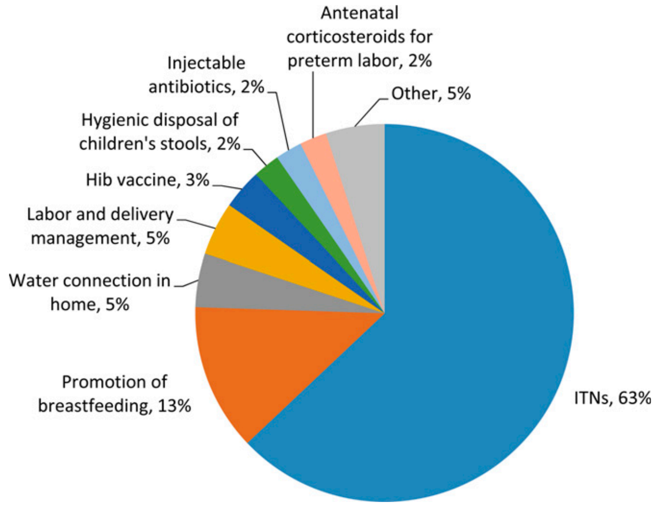

Supplemental Figure 2. Percentage of under-five mortality reduction in the comparison area by intervention, 2010–2013.

SUPPLEMENTAL WEB ANNEX 4: CONTEXTUAL FACTORS

Supplemental Table 1.4

Contextual factors in program and comparison areas, 2010–2013

	Program area		Comparison area	
	2010	2013	2010	2013
% Women 15–49 years with any education	9.2% (8.2–10.3%)	15.3% (13.9–16.7%)	11.1% (10.0–12.2%)	16.2% (14.7–17.9%)
% Women 15–49 years who migrated into their current province within the previous 3 years	NA	2.8% (2.5–3.3%)	NA	4.8% (4.2–5.3%)
% Population < 5 km from health facility[1,2]	47.1%	50.8%	49.7%	40.5%
First-level health facilities per 10,000 population[1,2]	1.11	1.18	1.17	1.10

Supplemental Table 2.4

Number of gold mining sites by region, 2010–2012[3]

	2010	2011	2012
Program regions			
Centre-Nord	49	34	37
Nord	30	18	19
Total	79	52	56
Regions containing comparison districts			
Centre-Est	9	11	12
Centre-Ouest	4	1	1
Centre-Sud	5	9	8
Est	30	30	30
Plateau Central	3	0	0
Total	51	51	51

SUPPLEMENTAL WEB ANNEX 5: REANALYSIS OF BURKINA FASO 2010 DEMOGRAPHIC AND HEALTH SURVEY (DHS) DATA BY MONTH

Supplemental Table 1.5

Stratification of careseeking indicators by month, Burkina Faso DHS 2010

Indicators	October 2010 (end of rainy season)		November—December 2010 (dry season)	
	%	95% CI	%	95% CI
Children with fever for whom care was sought	58.7	[41.3–74.1]	69.3	[60.9–76.7]
Children with suspected pneumonia for whom care was sought	78.7	[41.1–95.1]	82.3	[65.9–91.8]
Children with diarrhea for whom care was sought	51.2	[40.1–62.2]	56.5	[48.4–64.2]

Supplemental Table 2.5

Stratification of treatment indicators by month, Burkina Faso DHS 2010

Indicators	October 2010 (end of rainy season)		November–December 2010 (dry season)	
	%	95% CI	%	95% CI
Children with fever who received an antimalarial	32.9	[20.8–47.7]	35.4	[28.5–43.0]
Children with pneumonia who received an antibiotic	50.3	[26.8–73.6]	64.6	[45.6–79.9]
Children with diarrhea who received ORS	24.0	[13.0–40.1]	22.3	[14.6–32.5]

SUPPLEMENTAL REFERENCES

1. Direction Générale de l'information et des Statistiques Sanitaires du Ministère de la Santé, 2011. *Annuaire Statistique 2010*. Ouagadougou, Burkina Faso.
2. Direction Générale de l'information et des Statistiques Sanitaires du Ministère de la Santé, 2014. *Annuaire Statistique 2013*. Ouagadougou, Burkina Faso.
3. Burkina Faso Ministry of Mines and Energy, 2014. *2012 Statistical Handbook for the Ministry of Mines and Energy*. Ouagadougou, Burkina Faso.
